# Supplementary material for: A case-cohort longitudinal study for the analysis of microbial associations and viruses on the risk of celiac disease (MAVRiC)
Source: medRxiv. 2025 May 27:2025.05.26.25328184. Preprint. [Version 1] doi: 10.1101/2025.05.26.25328184 (PMC12148286; doi:10.1101/2025.05.26.25328184)
Supplement: Supplement 1 [file media-1.pdf]

Supplemental Figure 1

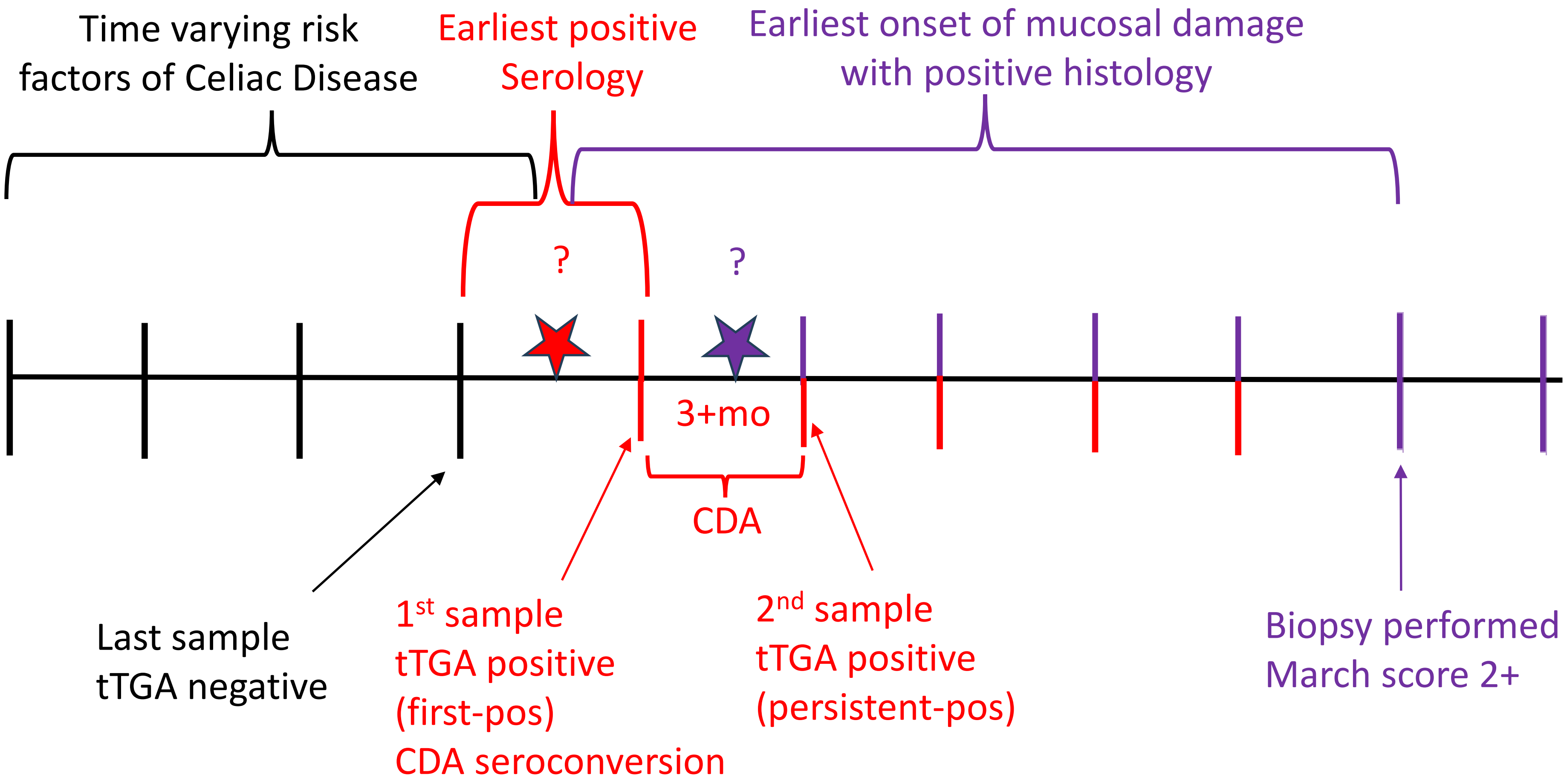

CD-onset = CDA seroconversion age < 4years followed by a positive biopsy

CD-hi = CDA seroconversion age <4 years with titer of persistent-positive sample ≥60 U/ml

Supplemental Figure 2

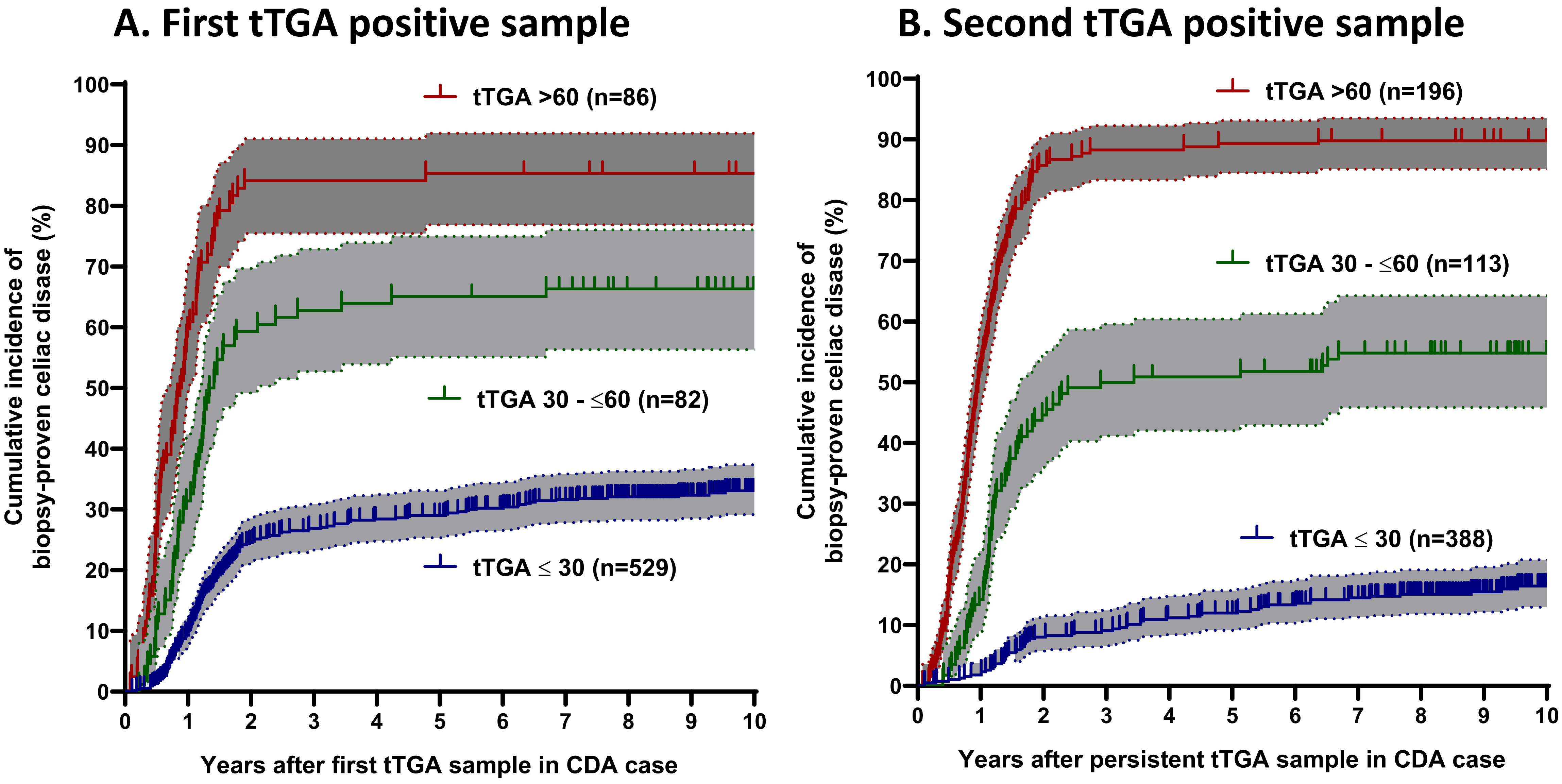

Supplemental Figure 3

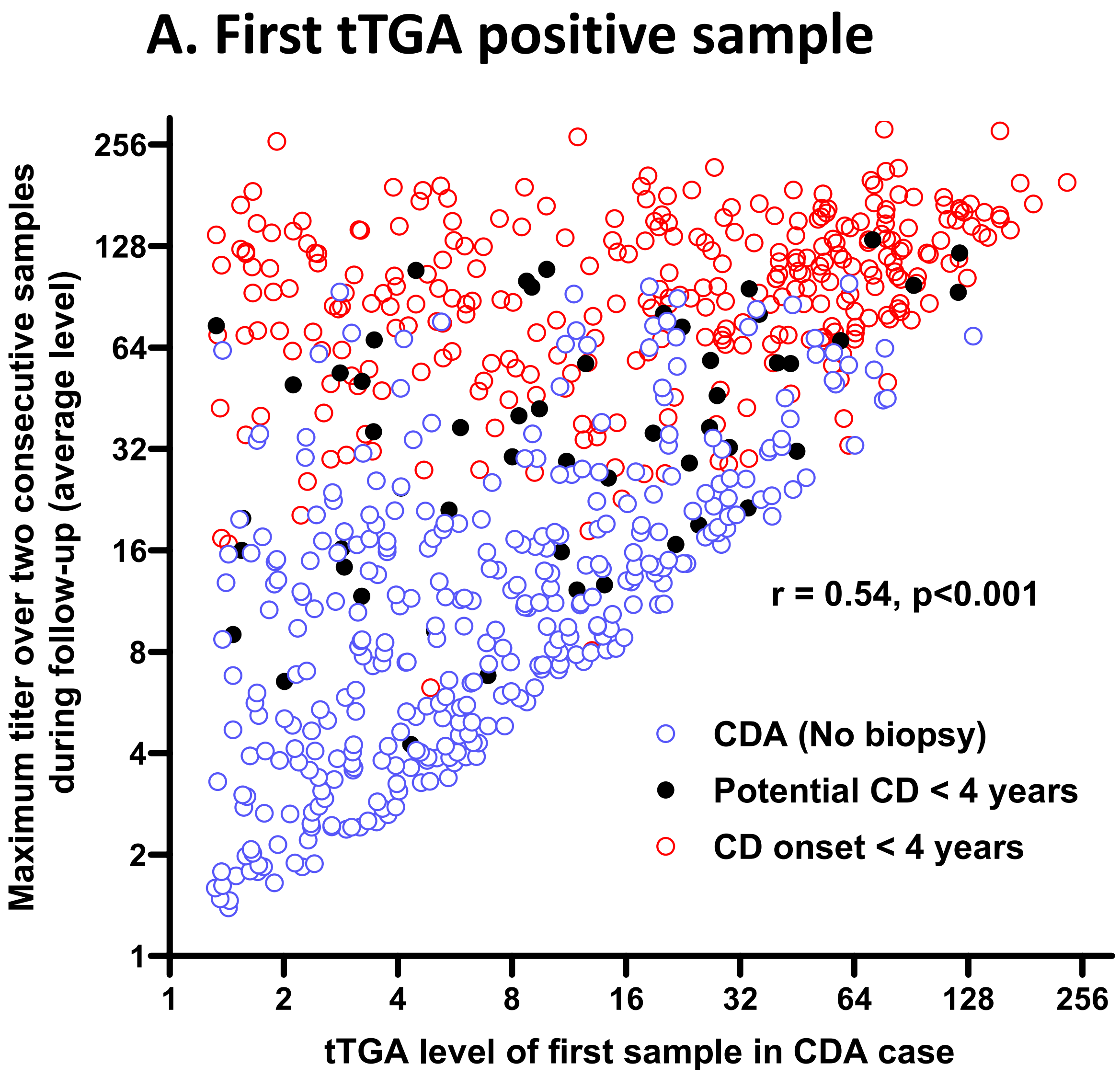

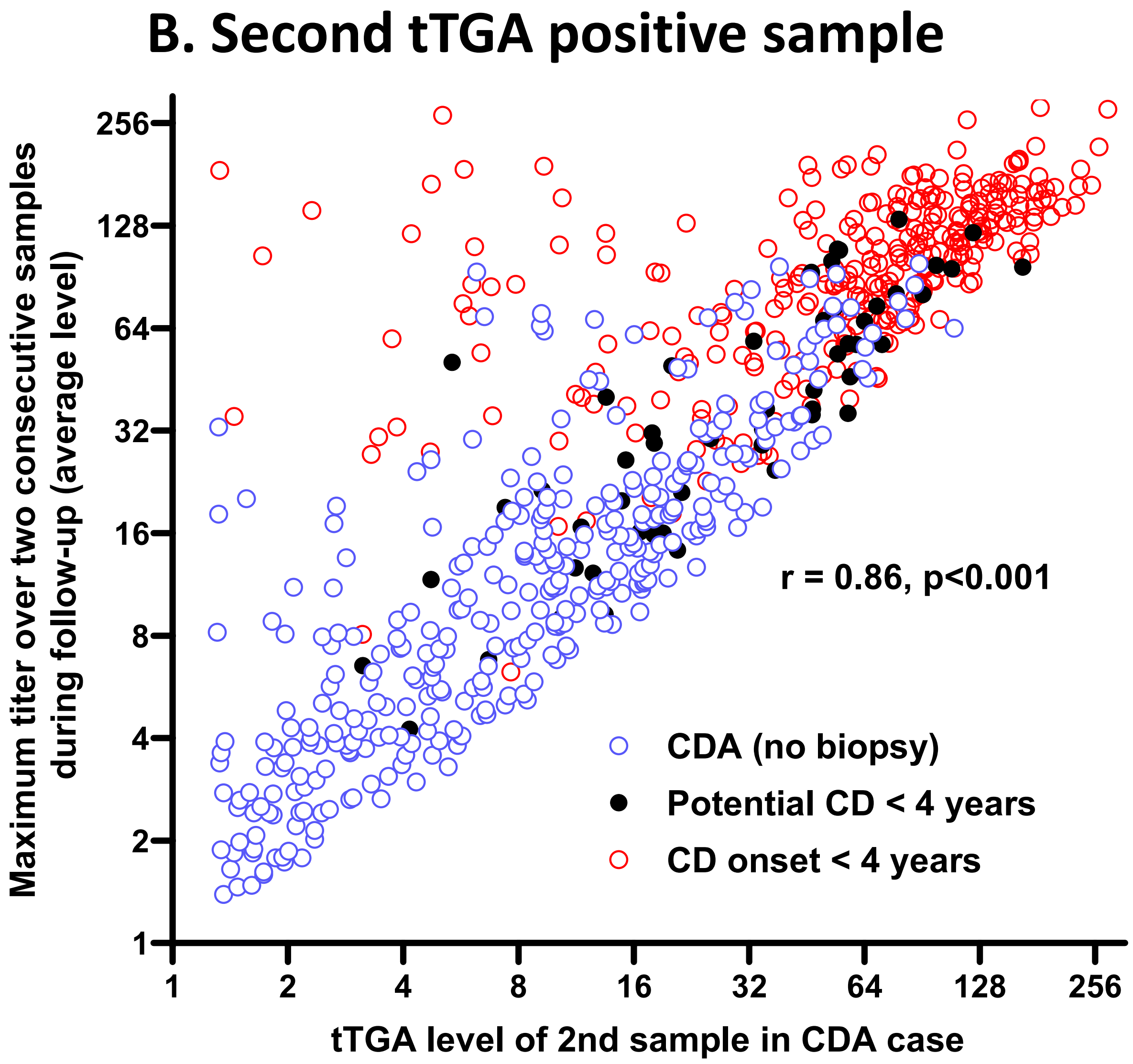

Supplemental Figure 4

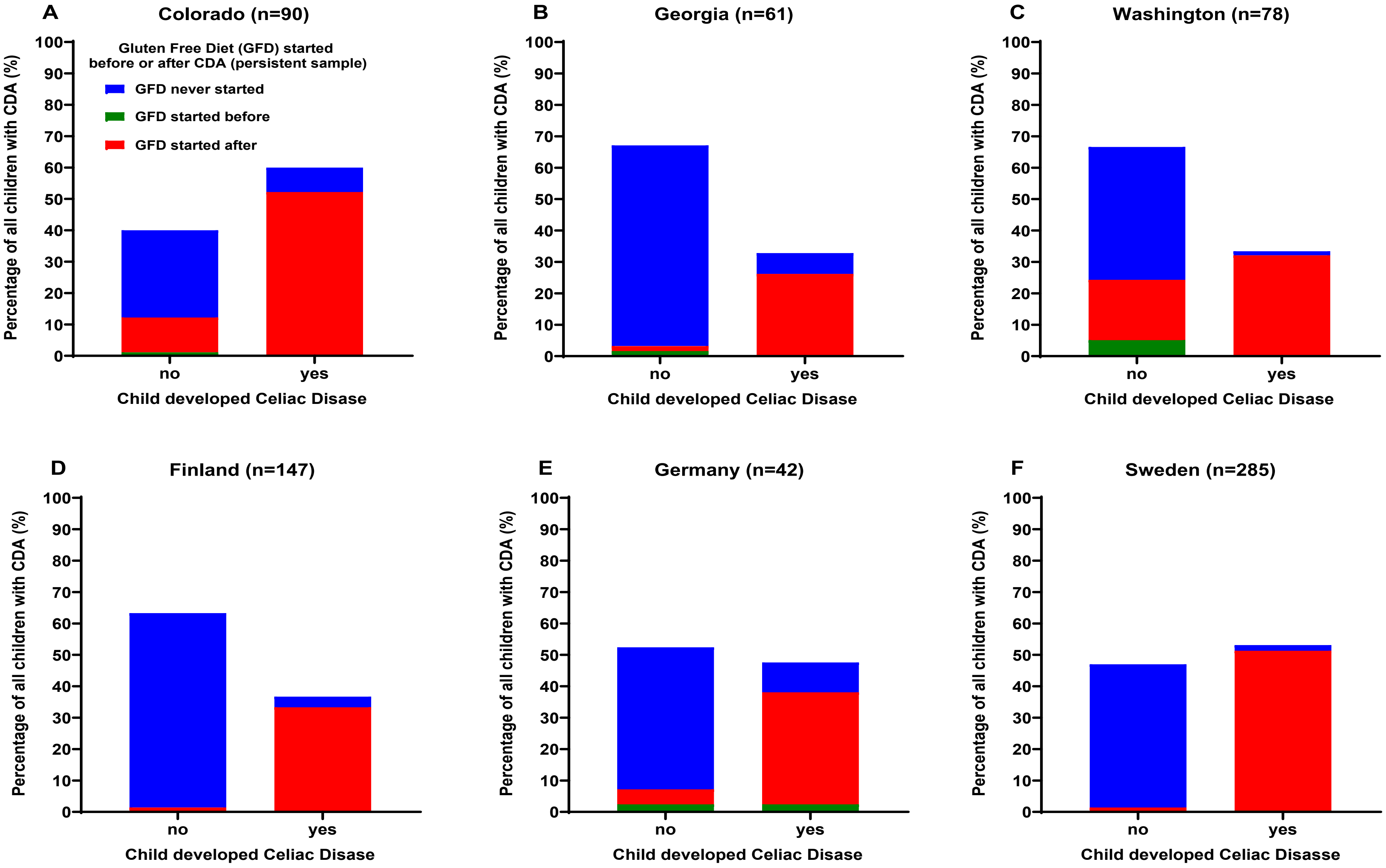

Supplemental table 1 SNPs used in the calculation of a non-HLA polygenic risk score (PRS) for the onset of celiac disease (CD-onset) before age 4-years.

SNP	Chromosome	Position (GRCh38)	Minor Allele [a]	Nearby Gene	log HR [b] (CD-onset)
rs72704176	1	155456067	T	ASHIL	0.5831
rs115195008	1	197649315	C	DENND1B	0.5324
rs3771689	2	159365291	T	BAZ2B	-0.3202
rs1829618	2	211568600	A	ERRB4	0.2669
rs12990970	2	203835966	T	CTLA-4	-0.1816
rs13014907	2	185208879	T	ZNF804A	0.7024
rs1464510	3	188394766	A	LPP	0.2671
rs6806528	3	69203748	T	FRMD4B	0.3886
rs12493471	3	45910186	T	CCR9	0.2200
rs114157400	4	102014304	G	BANK1	0.4529
rs1054091	6	159048480	C	TAGAP	0.3737
rs2327832	6	137651931	G	TNFAIP3	0.3137
rs802734	6	127957653	G	PTPRK	0.2901
rs61751041	7	107953740	T	LAMB1	0.6360
rs6967298	7	70549523	G	AUTS2	-0.3899
rs2409747	8	11220953	T	XKR6	0.3830
rs76554494	9	120628467	C	MEGF9	0.3314
rs9423406	10	5311727	A	AKR1C7P	0.2752
rs117561283	12	68052813	T	IFNG	0.5034
rs653178	12	111569952	C	SH2B3	0.1791
rs8013918	14	75242863	T	FOS	-0.2405
rs11203203	21	42416077	A	UBASH3A	0.2157
rs2298428	22	21628603	T	YDJC	0.3624

[a] = Forward strand based on the Genome Reference Consortium Human Build 38.

[b] = The PRS is created by summing across single nucleotide polymorphisms (SNPs) the product of the number of minor alleles by the log beta coefficient (log hazard ratio=log HR) that was estimated from a multivariate proportional hazard model on specific risk of CD-onset adjusting for country, number of HLA-DR3-DQ2 haplotypes, sex and first three principal components describing ancestry.
